# More capture, more suppression: Distractor suppression due to statistical regularities is determined by the magnitude of attentional capture

**DOI:** 10.3758/s13423-019-01672-z

**Published:** 2019-12-17

**Authors:** Michel Failing, Jan Theeuwes

**Affiliations:** 1 Department of Experimental and Applied Psychology, Vrije Universiteit Amsterdam, 1081 BT Amsterdam, The Netherlands; 2 Institute of Brain and Behavior Amsterdam, Amsterdam, The Netherlands; 3 Charité - Universitätsmedizin Berlin, Berlin, Germany; 4 Berlin Institute of Health, Berlin, Germany

## Abstract

Salient yet irrelevant objects often interfere with daily tasks by capturing attention against our best interests and intentions. Recent research has shown that through implicit learning, distraction by a salient object can be reduced by suppressing the location where this distractor is likely to appear. Here, we investigated whether suppression of such high-probability distractor locations is an all-or-none phenomenon or specifically tuned to the degree of interference caused by the distractor. In two experiments, we varied the salience of two task-irrelevant singleton distractors each of which was more likely to appear in one specific location in the visual field. We show that the magnitude of interference by a distractor determines the magnitude of suppression for its high-probability location: The more salient a distractor, the more it becomes suppressed when appearing in its high-probability location. We conclude that distractor suppression emerges as a consequence of the spatial regularities regarding the location of a distractor as well as its potency to interfere with attentional selection.

## Introduction

In everyday life, we try to attend to what is relevant to us but the visual world provides an overload of input that necessitates selective filtering and attenuation (Broadbent, 1958). Attentional selection is traditionally thought to be driven by competitive advantages (Desimone & Duncan, 1995) that arise from either strategic top-down processes (Leber & Egeth, 2006) or bottom-up processes that automatically bias attention towards physically measurable stimulus features (e.g., color, luminance, etc.) that are salient relative to their local surroundings (Theeuwes, 2010).

The salience of a stimulus has a particularly straightforward relationship with the extent to which that stimulus attracts attention. For instance, Theeuwes (1992) manipulated the physical salience of a distractor and showed that the more salient the distractor, the more it captured attention. Similarly, Donk and colleagues (Donk & van Zoest, 2008; van Zoest, Donk, & Theeuwes, 2004) demonstrated that the degree to which orientation distractors deviated from their local surroundings determined to which extent they interfered with overt target selection. Distractors with larger orientation differences to the surrounding stimuli led to a smaller proportion of first eye movements landing at the target compared to distractors with relatively smaller orientation differences. These findings fit well with attention models that incorporate a priority map of attentional selection. This map is a topographic representation of attentional selection priority according to which the representation of visual space with the largest activity will determine which location in space will be attended (Fecteau & Munoz, 2006; Itti & Koch, 2001; Theeuwes, 2018). Within this framework, parametric manipulations of distractor salience are represented as stronger or weaker activity for the spatial location of the distractor on the priority map.

Recent findings highlighted that past episodes of selection have a significant influence on attention above and beyond the influence of top-down or bottom-up control due to implicit and automatic learning processes (Awh, Belopolsky, & Theeuwes, 2012; Failing & Theeuwes, 2018; Theeuwes, 2018). As a consequence of experienced regularities in spatial (Chun & Jiang, 1998; Geng & Behrmann, 2005), feature (Maljkovic & Nakayama, 1994), or temporal information (Zhao, Al-Aidroos, & Turk-Browne, 2013), such learning processes, or visual statistical learning (VSL; Turk-Browne, Junge, & Scholl, 2005), lead to lasting attentional biases towards the features bearing the regularities (Failing & Theeuwes, 2018; Theeuwes, 2018). While previous studies showed that VSL influences what is more likely to be attended, most recent studies suggest that VSL also has bearing on what is more likely to be avoided. For example, it was shown that presenting a salient distractor more often in a particular location in space leads to the spatial suppression of that location (Ferrante et al., 2018; Wang & Theeuwes, 2018a, 2018b). This suppression is typically not the consequence of explicit expectations or search strategies of the observers as most remain completely unaware of the spatial regularities. Subsequent studies showed that VSL regarding task-relevant and task-irrelevant regularities impacts attention differently, and extends to learning about regularities at multiple locations and other visual features of distractors (e.g., color or shape; Failing, Wang, & Theeuwes, 2019a; Failing et al., 2019b; Stilwell, Bahle, & Vecera, 2019).

It has recently been argued that VSL-induced suppression alters the activity profile on the priority map much like top-down or bottom-up attentional signals (Failing & Theeuwes, 2018; Theeuwes, 2018). However, it is unclear whether the suppressive modulations follow an all-or-none principle or manifest themselves more adaptively in close relation to the need of suppression that arose from the visual input during VSL. More specifically, is suppression adaptive such that the more salient a distractor the more suppression is applied; or, alternatively, is suppression always applied in an all-or-none fashion irrespective of the saliency signal that needs to be suppressed. Because task-irrelevant distractors can be more or less interfering depending on their objectively measurable saliency (Theeuwes, 1992; van Zoest et al., 2004), adaptively applying suppression would seem the most efficient mechanism. However, an adaptive suppression mechanism might also be more effortful for the visual system than an all-or-none mechanism because there is the need to constantly track potential differences in the saliency signal.

The present study was designed to investigate whether the suppression at high-probability distractor locations is an all-or-none phenomenon or varies adaptively with the saliency signal present at the to-be-suppressed location. To this end, participants searched for a singleton target in two experiments while ignoring either a color (Experiment 1) or luminance singleton distractor (Experiment 2) that was either more or less salient. To directly compare the extent to which salience affects suppression due to regularities regarding the distractor position, the high-salience distractor was more likely to appear in one location and the low-salience distractor was more likely to appear in another location. Yet, each of the distractor types was equally often presented across all trials. We hypothesized that if distractor salience determines the extent of suppression at high-probability distractor locations, suppression applied to these locations should differ depending on the saliency of the signal that is most likely to be presented at that location. High probability locations of highly salient distractors should thus receive larger suppression than high-probability locations of low salient distractors.

## Methods

### Experiment 1 and 2

#### Participants

Twenty-four volunteers (21 female) participated in Experiment 1 and 24 other volunteers (14 female) participated in Experiment 2. All participants had reported normal or corrected-to-normal vision and were naïve as to the study’s purpose.

#### Apparatus and stimuli

All stimuli in both experiments were created using OpenSesame (Mathôt, Schreij, & Theeuwes, 2012) and presented on a uniform black background (~0 cd/m^2^) at a distance of 75 cm. A gray fixation dot (~22 cd/m^2^) was visible throughout each trial.

##### Experiment 1

The search display contained eight outline shapes that were always of one color (chosen from red and green; fixed for an individual but counterbalanced across all participants). However, depending on the condition, one of the non-target shapes could be of the other color (i.e., either red or green depending on the counterbalancing schedule) or a yellowish color. All colors were approximately isoluminant (~14 cd/m^2^).

All shapes were presented on an imaginary circle at equal distance (4.75 visual degrees radius). Each search display contained one shape singleton that was either a diamond (1.35°) among circles (1.1°) or vice versa. Within each shape was a gray line segment (0.75°; ~22 cd/m^2^) that had either one of two orientations (horizontal or vertical).

##### Experiment 2

The search display was identical to Experiment 1 except that all outline shapes were dark gray outline shapes (~9 cd/m^2^). Depending on the condition, one of the shapes could have a brighter shade of gray (~37 cd/m^2^) or be white (~103 cd/m^2^).

#### Procedure and design

##### Experiment 1

**Fig. 1:**
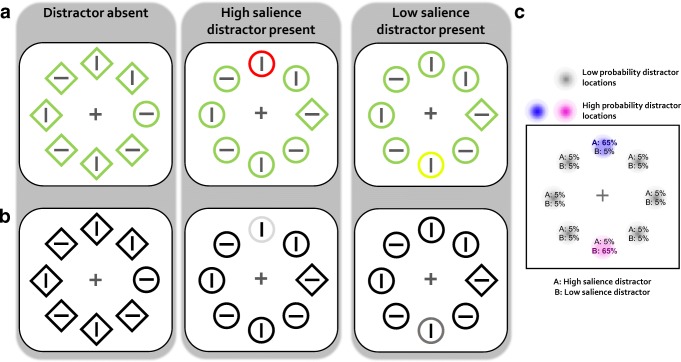
(**a**) Search display examples for Experiment 1. Illustration shows three different conditions: distractor-absent, high-salience distractor-present, and low-salience distractor-present conditions. Participants had to search for a shape singleton and, on distractor-present trials, ignore a color singleton distractor. The distractor in one color (e.g., red) was more likely to appear in one position along the imaginary circle (e.g., top position), while the distractor in another color (e.g., yellowish) was more likely to appear in another position (e.g., bottom position). (**b**) Search display examples for Experiment 2 showing the same conditions as in (**a**). Participants performed the same task as in Experiment 1 but had to ignore a luminance singleton on distractor-present trials. The distractor in one luminance intensity was more likely to appear in one position along the imaginary circle (e.g., top position), while the distractor in another luminance intensity was more likely to appear in another position (e.g., bottom position). Note that the background in the actual experiments was black and is only shown in white here for illustrative purposes. (**c**) Schematic representation of the spatial and salience regularities of the distractor. The two high-probability distractor locations are shown in purple and pink, while the low-probability locations are shown in gray. Percentages at each location represent the probabilities of each distractor type to appear in a given location.

The fixation display showed a fixation cross for 700–1,000 ms followed by a search array presented for 1,500 ms or until response. Participants searched for a shape singleton (“target”) and responded to the orientation of the line segment inside the target. Two design features were critical to this experiment: First, on one-sixth of all trials, all shapes had the same color (e.g., green; distractor-absent trials; Fig. 1A). On the remaining trials (distractor-present trials), one of the non-target shapes (“distractor”) had a different color that could either be a high-salience (e.g., red) or a low-salience (“yellowish”) color. Note that this means that the target color remained the same in each trial and that there could always only be a single distractor present in any given distractor present trial. Color salience of the distractor was determined on the basis of distance between colors in CIE color space while keeping the luminance identical. A pilot experiment confirmed that the high-salience distractor elicited more attentional capture than the less salient distractor (for more details on the pilot experiment, see Supplemental Material). Second, each distractor type was more likely (65% probability) to appear in one of the eight positions within the search display with the constraint that both of the high-probability locations were at maximum distance from each other (i.e., on opposite sides of the imaginary circle; counterbalanced across participants). For one participant, for example, the high-salience distractor was more probable at the top position, while the low-salience distractor was more probable at the bottom position. Each distractor type was equally likely to appear at all of its remaining low-probability locations (5% probability; see Fig. 1C). This resulted in three distractor position conditions: high-probability distractor location of the high-salience distractor, high-probability distractor location of the low-salience distractor and low-probability distractor location.

Each participant performed one practice block of 20 trials and 12 experiment blocks of 192 trials each. After the experiment, each participant had to fill in an implicit learning questionnaire querying them with three questions about the distractors and their spatial regularities. For the first question, participants were informed that the distractors displayed certain regularities and were asked to indicate which, if any, they had noticed. For the last two questions, they were explicitly told that the high-salience distractor was more likely to appear in one while the low-salience distractor was more likely to appear in another location, and then asked to indicate these locations for each distractor separately.

##### Experiment 2

Experiment 2 was basically identical to Experiment 1. However, all stimuli of the search display were dark gray, except on distractor-present trials in which one of the shapes was a luminance singleton (Fig. 1B). This could either be a high-luminance (white) or a low-luminance distractor (gray). We will refer to these as high- and low-salience distractors. Each of the distractors was more probable to appear in a specific location leading to the exact same distractor position conditions as in Experiment 1: high-probability distractor location of the high-salience distractor, high-probability distractor location of the low-salience distractor and low-probability distractor location.

## Results

For the analyses of average response times (RTs), incorrect and responses faster than 200 ms (< 1% in both experiments) were discarded.

### Experiment 1

#### Attentional capture

To demonstrate that the distractor interfered with target search and, crucially, that the interference varied with its salience, we first submitted RT data to an ANOVA with distractor presence (absent vs. high-salience distractor vs. low-salience distractor) as factor. This effect was reliable (*F*(2,46)=57.87, *p*<.001, ηp^2^=.716). Planned comparisons revealed that both distractors interfered with target search (absent vs. high-salience distractor: *M*=825 ms ± *SD*=92 vs. 856 ms ± 95, *t*(23)=10.437, *p*<.001, Cohen’s *d*=2.130; absent vs. low-salience distractor: 825 ms ± 92 vs. 844 ms ± 92 *t*(23)=7.256, *p*<.001, *d*=1.481). Crucially, the high-salience distractor caused larger interference than the low-salience distractor (*t*(23)=3.785, *p*<.001, *d*=.773). Similar results were obtained for error rates with all effects being congruent with (i.e., the same direction as) the RT effects (*p*<.01), which excludes an alternative explanation in terms of a speed-accuracy trade-off. This demonstrates that our salience manipulation was successful, suggesting that while both distractors captured attention, there was more capture by the high- than by the low-salience distractor.

#### Attentional suppression

**Fig. 2:**
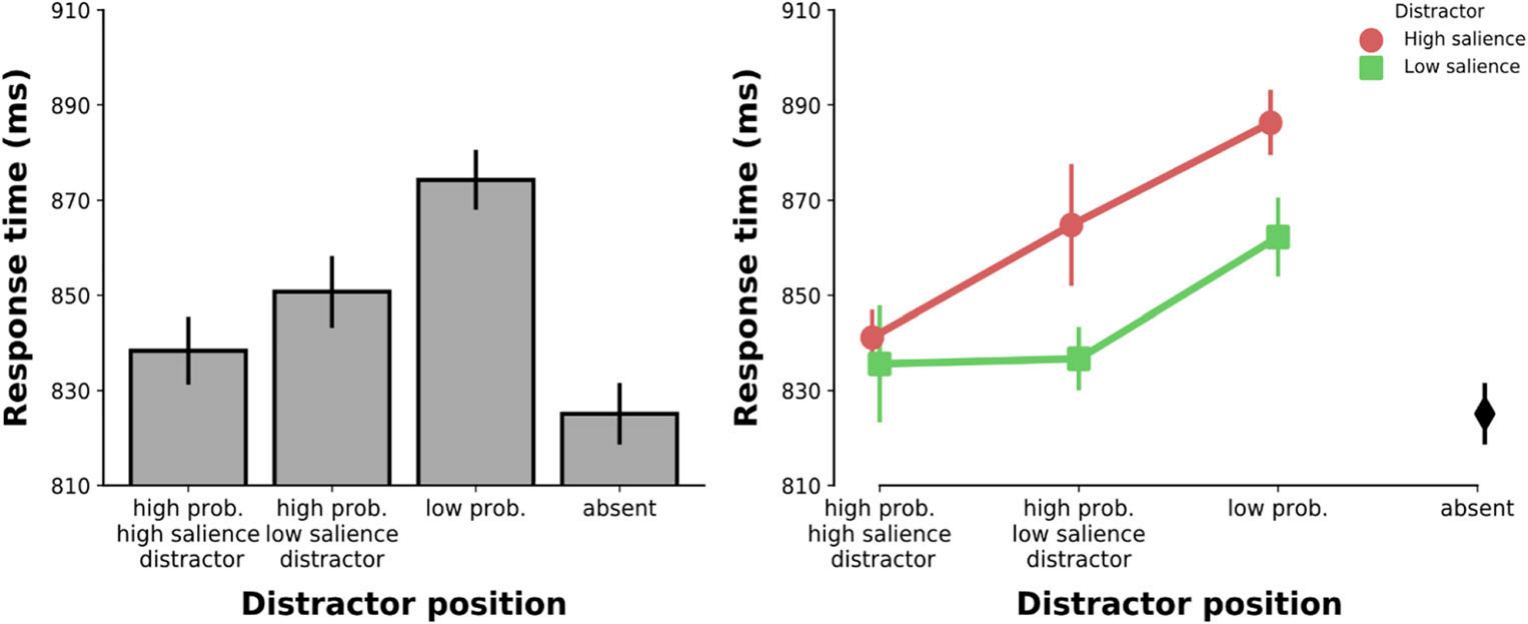
Results of Experiment 1. **Left panel:** Mean response time by distractor position condition. **Right panel:** Mean response time by distractor salience over distractor position condition. Error bars here, and in all the following figures, represent 95% within-subject confidence intervals (Cousineau, 2005; Morey, 2008)

To investigate the impact of our probability manipulation on the distractor’s interference, we assessed whether search times differed depending on where any of the distractors appeared. Relative to distractor-absent trials, RTs were significantly slower when a distractor appeared in the high-probability location of the high-salience distractor (*t*(23)=3.071, *p*=.005, *d*=1.060) or the low-salience distractor (*t*(23)=6.953, *p*<.001, *d*=1.419; see Table 1 for RT and error rates), or one of the low-probability locations (*t*(23)=12.854, *p*<.001, *d*=2.624; Fig. 2, left). Both high-probability distractor locations showed evidence for suppression since target search was significantly faster when a distractor appeared in one of the high-probability locations relative to when it appeared in a low-probability location (vs. high-salience distractor location: *t*(23)=10.532, *p*<.001, *d*=2.150; vs. low-salience distractor location: *t*(23)=5.337, *p*<.001, *d*=1.089). Crucially however, target search was even faster when a distractor appeared in the high-probability location of the high-salience distractor relative to when it appeared in the high-probability location of the low-salience distractor (*t*(23)=2.656, *p*=.014, *d*=.542). There was no evidence for a speed-accuracy trade-off (all comparisons either *p*>.1 or congruent with the RT effects when significant). These findings suggest that the high-probability distractor locations were suppressed differently: the high-probability location of the high-salience distractor was suppressed more strongly than the high-probability location of the low-salience distractor.

**Table 1:** Mean response times (RTs) and error rates of both experiments

Experiment	Distractor type	Distractor location	RT (in ms)	Error rate (in percent)
1	Any distractor	HPL of high-salience distractor	838 (98)	13.5 (5.9)
		HPL of low-salience distractor	851 (96)	13.7 (5.9)
		LPL	874 (96)	15.9 (6.6)
	High salience	HPL of high-salience distractor	841 (93)	14.1 (5.8)
		HPL of low-salience distractor	865 (105)	14.5 (7.3)
		LPL	886 (99)	16.3 (7.1)
	Low salience	HPL of high-salience distractor	836 (106)	12.9 (7.0)
		HPL of low-salience distractor	837 (90)	12.9 (5.2)
		LPL	862 (94)	15.5 (6.4)
2	Any distractor	HPL of high-salience distractor	751 (108)	7.4 (4.6)
		HPL of low-salience distractor	761 (113)	7.0 (5.1)
		LPL	784 (112)	7.8 (4.2)
	High salience	HPL of high-salience distractor	755 (109)	7.2 (3.8)
		HPL of low-salience distractor	776 (115)	7.3 (7.2)
		LPL	799 (115)	7.6 (4.1)
	Low salience	HPL of high-salience distractor	747 (108)	7.6 (5.7)
		HPL of low-salience distractor	746 (111)	6.8 (3.6)
		LPL	769 (111)	8.0 (4.5)

Data between parentheses represent standard deviations

*HPL* high-probability location, *LPL* low-probability location

Next, we examined whether the pattern of suppression differed depending on which of the two distractors was present. An ANOVA on mean RT distractor salience (high vs. low) and distractor position (high-probability location of high-salience distractor vs. high-probability location of low-salience distractor vs. low-probability location) as factors showed a main effect of distractor salience (*F*(2,23)=22.999, *p*<.001, ηp^2^=.500), distractor position (*F*(2,46)=42.187, *p*<.001, ηp^2^=.647), as well as a significant interaction (*F*(2,46)=5.459, *p*=.007, ηp^2^=.192). In line with our previous analysis, presenting either distractor in either its high-probability location or the high-probability location of the other distractor significantly reduced its interference with target search relative to when it appeared in a low-probability location (all comparisons *p*<0.001; Fig. 2, right). However, only when the high-salience distractor appeared in its specific high-probability location, was interference in target search even further reduced (high-salience distractor trials: high-probability location of high-salience distractor vs. high-probability location of low-salience distractor: *t*(23)=3.665, *p*=.001, *d*=.748). No such difference was observed for the low-salience distractor (low-salience distractor trials: high-probability location of high-salience distractor vs. high-probability location of low-salience distractor: *t*(23)=.180, *p*=.859). Although interference by the two distractors did not differ for the high-probability location of the high-salience distractor (*t*(23)=.879, *p*=.389), interference by the low-salience distractor when it appeared at this location was so far reduced as to be statistically indistinguishable from search performance in distractor-absent trials (*t*(23)=1.503, *p*=.146). Finally, there was no evidence for a speed-accuracy trade-off as all comparisons were either non-significant (*p*>.1) or congruent with RT effects when significant.

#### Intertrial priming

To assess whether suppression can be explained in terms of short-lived, intertrial location priming instead of learning about the statistical regularities regarding the distractor positions, we compared trials in which the distractor position of a given trial was identical to the previous trial with trials in which it had changed (same position vs. different position). Indeed, there was evidence for intertrial location priming as RT (*t*(23)=8.006, *p*<.001, *d*=1.634) and error rate (*t*(23)=3.352, *p*=.003, *d*=1.124) in same-position trials was lower than in different-position trials. Importantly though, intertrial location priming could neither explain the overall suppression effects nor the differences in suppression between the two distractors in particular. After excluding all trials in which the distractor position was repeated between trials (i.e., same-position trials), an ANOVA on RT with distractor salience (high vs. low) and distractor position (high-probability location of high-salience distractor vs. high-probability location of low-salience distractor vs. low-probability location) replicated all major findings. There was no evidence for a speed accuracy trade-off (all comparisons non-significant (ns) at *p*>.1 or congruent with the RT effects).

Using a similar approach, we also investigated whether the suppression effects can be explained in terms of intertrial feature priming. To this end, we compared trials in which the distractor type of a given trial was identical to the distractor type in the previous trial (same-distractor vs. different-distractor type). This difference reached significance for RT (*t*(23)=2.227, *p*=.036, *d*=.455) but not for error rate (*t*(23)=1.023, *p*=.317, *d*=.209). Importantly though, after excluding all trials in which the distractor type repeated between trials, the ANOVA replicated all major findings, which shows that intertrial feature priming had no influence on the observed suppression effects. There was no evidence for a speed accuracy trade-off (all error rate comparisons *p*>.1).

#### Spatial gradient of suppression

**Fig. 3:**
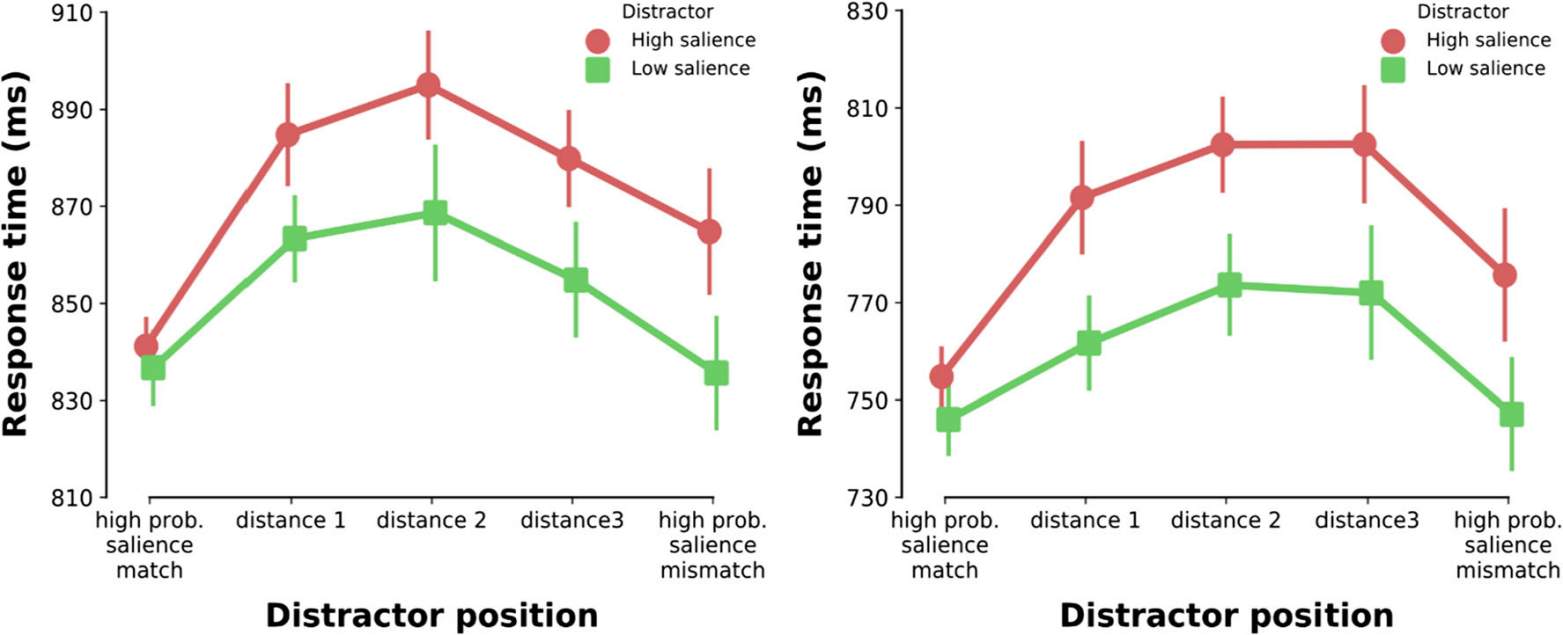
Results of Experiment 1 and Experiment 2. **Left panel:** Mean response time by distractor salience over distractor position. Data are shown beginning with trials in which the salient color singleton distractor appeared in the high-probability location that matched its salience to trials in which it appeared in the high-probability location that did not. Data are collapsed across conditions in which the distractor presentation was symmetric along the vertical meridian. **Right panel:** The same analysis for Experiment 2

We also analyzed the spatial profile of suppression by assessing how interference by the distractor changed as a function of the distractor’s distance to the high-probability distractor locations (indexed by the number of positions). An ANOVA on RT with distractor salience (high vs. low) and distance of the distractor to the high-probability location matching its salience (from distance 0, or high-probability location salience match, to distance 4, or high-probability location salience mismatch) as factors showed a main effect of distractor salience (*F*(1,23)=30.647, *p*<.001, ηp^2^=.571) and distance (*F*(4,92)=22.969, *p*<.001, ηp^2^=.500) as well as a significant interaction (*F*(4,92)=2.515, *p*=.047, ηp^2^=.099). Figure 3 (right panel) shows a clear spatial gradient pattern for both distractor types mirroring the key features of the previous analyses. The spatial gradient for the distractors followed a quadratic trend (*t*(23)=9-234, *p*<.001), albeit the gradient for the high-salience distractor showed a steeper rise from its salience-matching high-probability location. There was no evidence of a speed-accuracy trade-off in the error rates (all comparisons *p*>.1 or congruent with the RT effects). These results provide strong evidence for a spatial gradient in the suppression of high-probability distractor locations: the closer a given distractor to a high-probability location, the stronger the suppression.

#### Awareness

Out of 24 participants, six indicated in the implicit learning questionnaire that they had noticed some relationship. Two participants correctly identified both high-probability locations (chance level ~1.6% or zero participants), although these two participants had indicated that they had noticed no relationship. Removing those two participants had no significant influence on the main findings.

### Experiment 2

#### Attentional capture

An ANOVA on RT with distractor presence (absent vs. high-salience distractor vs. low-salience distractor) as factor showed a significant main effect (*F*(2,46)=56.24, *p*<.001, ηp^2^=.710). Both distractors interfered with target search (absent vs. high-salience distractor: 726 ms ± 104 vs. 769 ms ± 111, *t*(23)=8.367, *p*<.001, *d*=1.708; absent vs. low-salience distractor: 726 ms ± 104 vs. 753 ms ± 110, *t*(23)=7.890, *p*<.001, *d*=1.611), but interference by the high-salience distractor was stronger (*t*(23)=4.564, *p*<.001, *d*=.932). There were no significant differences in error rate, which excludes the possibility of a speed-accuracy trade-off (all *p*>.1). This suggests that the distractor captured attention and that capture by the high-salience distractor was larger.

#### Attentional suppression

**Fig. 4:**
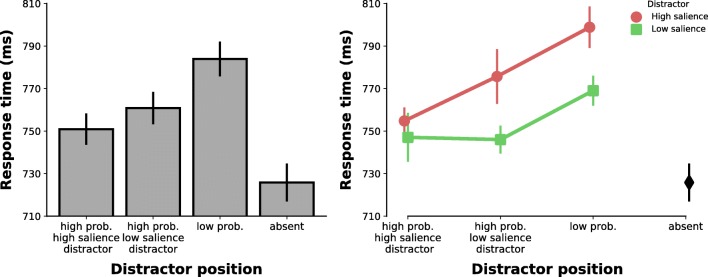
Results of Experiment 2. **Left panel:** Mean response time by distractor-position condition. **Right panel:** Mean response time by distractor salience over distractor-position condition

The distractor interfered significantly with target search no matter where it appeared (Fig. 4, left; absent vs. high-probability location of high-salience distractor, *t*(23)=4.913, *p*<.001, *d*=1.003, vs. high-probability location of low-salience distractor, *t*(23)=7.931, *p*<.001, *d*=1.619, vs. low-probability location, *t*(23)=10.632, *p*<.001, *d*=2.170; see Table 1 for RT and error rates). As expected, when the distractor appeared in either high-probability location it interfered less compared to when it appeared in a low-probability location (vs. high-salience distractor location: *t*(23)=8.380, *p*<.001, *d*=1.711; vs. low-salience distractor location: *t*(23)=4.771, *p*<.001, *d*=.974). When any distractor appeared in the high-probability location of the high-salience distractor, interference by the distractor was even further reduced (high vs. low-salience distractor location: *t*(23)=2.160, *p*=.041, *d*=.441). All comparisons on error rate were either non-significant (*p*>.1) or congruent with RT effects. Similar to Experiment 1, these findings suggest that suppression was different for the two high-probability distractor locations. The high-probability location containing the high-salience distractor was suppressed more strongly than the high-probability location containing the low-salience distractor.

An ANOVA on RT with distractor salience (high vs. low) and distractor position (high-probability location of high-salience distractor vs. high-probability location of low-salience distractor vs. low-probability location) showed a main effect of distractor salience (*F*(2,23)=40.402, *p*<.001, ηp^2^=.637), distractor position (*F*(2,46)=29.159, *p*<.001, ηp^2^=.559), and a significant interaction (*F*(2,46)=4.742, *p*=.025, ηp^2^=.171). Confirming the previous analysis, when a distractor appeared either in its high-probability location or the high-probability location of the other distractor, its interference on target search was significantly reduced (all comparisons *p*<.01; Fig. 4, right). However, similar to Experiment 1, only when the high-salience distractor appeared in its high-probability location was interference even further reduced (high-salience distractor trials: high-probability location of high-salience distractor vs. high-probability location of low-salience distractor: *t*(23)=3.254, *p*=.003, *d*=.664). This was not the case for the low-salience distractor (low-salience distractor trials: high-probability location of high-salience distractor vs. high-probability location of low-salience distractor: *t*(23)=.162, *p*=.872). Interference at the high-probability location of the high-salience distractor did not differ between the two distractors (*t*(23)=1.313, *p*=.202), but target search was significantly impaired relative to distractor-absent trials for both distractors (both *p*<.01). Finally, there was no evidence for a speed-accuracy trade-off (all comparisons either ns at *p*>.1 or congruent with RT effects at *p*<.05).

#### Intertrial priming

Similar to Experiment 1, we also found evidence for intertrial location priming when comparing RT (*t*(23)=8.823, *p*<.001, *d*=1.801) and error rate (*t*(23)=2.198, *p*=.038, *d*=.449) of same-position against different-position trials. Nonetheless, after removing priming (i.e., same-position) trials, an ANOVA on RT with distractor salience (high vs. low) and distractor position (high-probability location salience match vs. high-probability location salience mismatch vs. low-probability location) replicated all previous effects and showed no evidence of a speed-accuracy trade-off (all error rate comparisons *p*>.1).

In Experiment 2, we did not find any evidence for intertrial feature priming when comparing either RT (same vs. different distractor type: *t*(23)=1.615, *p*=.12, *d*=.33) or error rate (*t*(23)=.585, *p*=.564, *d*=.119). Removing trials in which the distractor type repeated between trials also had no influence on the major findings of this experiment, as revealed by the ANOVA on RT with distractor salience and distractor position as factors. There was also no evidence for a speed-accuracy trade-off (all error rate comparisons *p*>.1).

#### Spatial gradient of suppression

The analysis on the spatial gradient of suppression showed a main effect of distractor salience (*F*(1,23)=49.936, *p*<.001, ηp^2^=.685) and distance (*F*(4,92)=18.647, *p*<.001, ηp^2^=.448) as well as a marginally significant interaction (*F*(4,92)=2.198, *p*=.075, ηp^2^=.087). There was a spatial gradient pattern for both distractor types that mirrored the key aspects of the previous analyses (Fig. 3, right). Similar to Experiment 1, the spatial gradient for the distractors followed a quadratic trend (*t*(23)=8.145, *p*<.001) with yet again a steeper rise for the high-salience distractor condition from the distractor’s salience-matching high-probability location. There were no significant differences in error rates (all *p*>.1). In short, there is clear evidence for a spatial gradient of suppression for the two high-probability distractor locations with suppression centered around the high-probability locations and gradually falling off the further away from those locations.

#### Awareness

Out of 24 participants, ten indicated in the implicit learning questionnaire that they had noticed some relationship. However, not a single participant correctly identified both high-probability locations (chance level ~1.6% or zero participants).

## Discussion

In two experiments, we showed that distractor suppression at high-probability distractor locations is adaptive, such that the more salient a distractor, the more suppression is applied. Previous studies have shown that locations that are more likely to contain a distractor are suppressed relative to other locations (Ferrante et al., 2018; Wang & Theeuwes, 2018a, 2018b). The current study shows that the strength of suppression applied depends on the salience of the distractor that is more likely to be presented at that location. If a location was more likely to contain a highly salient distractor it was suppressed more strongly than a location that was more likely to contain a less salient distractor. This modulation of location-specific suppression was observed for two feature dimensions, color (Experiment 1) and luminance (Experiment 2), suggesting that it is feature-independent.

Even though our findings are generally consistent with the signal suppression hypothesis, which states that capture by irrelevant feature singletons can be avoided through selective inhibition (Gaspelin, Leonard, & Luck, 2015; Sawaki & Luck, 2010), they are inconsistent with a recent proposition according to which suppression occurs through first-order feature suppression (i.e., only suppression of feature values is possible; Gaspelin & Luck, 2018). The modulations in suppression we observed as a consequence of differences in salience between the two distractors is only explainable by incorporating differences between the feature value of the distractor *and* its nearby objects. As such our results are more consistent with evidence for second-order feature suppression models (Sawaki & Luck, 2010; Won et al., 2019). Although we note that Gaspelin and Luck acknowledged that second-order suppression cannot be ruled out, it does not appear from our findings that first-order suppression is more powerful. Indeed, if first-order suppression would have taken precedence, there should have been equally strong suppression at both high-probability distractor locations. Nonetheless, our findings also do not exclude the possibility that first-order suppression is possible under other circumstances. Future research should assess the contribution and boundary conditions from each of the suppression types.

As we have proposed earlier (e.g., Failing et al., 2019), suppression of high-probability distractor locations may be the result of an accumulation of a sufficient number of memory traces of suppression. This idea is inspired by instance theories of automatic behavior and attention (Logan, 1998, 2002), which stipulate learning through the accumulation of separate memory traces with experience. Memory traces that lead to suppression might be formed by past episodes of bottom-up selection of the salient distractor and the subsequent need to suppress them in order to re-orient attention to the target. The priority in attentional processing of the distractor due to bottom-up capture might thereby play an important role in the accumulation of memory traces because without attention there is considerably less learning (e.g., Logan & Etherton, 1994; Nissen & Bullemer, 1987). This notion is supported by other studies that highlight the importance of (initial) attentional prioritization of stimuli or stimulus locations that bear statistical regularities to elicit VSL-induced attentional biases (Failing & Theeuwes, 2017; Zhao et al., 2013). The current study extends this proposal by providing evidence for the idea that memory traces also represent the strength of suppression that was necessary during learning.

Our results suggest that the more salient a distractor the more suppression is applied to the high-probability location of that distractor. This is particularly evident for the high-salience distractor that is more strongly suppressed at the high-probability location of the high-salience distractor compared to the high-probability location of the low-salience distractor. However, we did not observe this difference for the low-salience distractor. That is, even though interference by the low-salience distractor was reduced when it appeared in either high-probability location, there was no additional reduction in interference when it appeared in the high-probability location of the high-salience distractor. The explanation for this result is not immediately clear. One explanation for this lack of “extra” suppression might be a ceiling effect. Indeed, additional suppression of the low-salience distractor at the high-probability location of the high-salience distractor would likely be equivalent to performance at or even below baseline levels (i.e., distractor absent condition; see Figs. 2 and 4, right panel). Previous studies investigating suppression as a consequence of regularities regarding the distractor location have, however, suggested that suppression cannot prevent residual capture by the distractor (e.g., Failing et al., 2019; Failing, Wang, & Theeuwes, 2019b; Ferrante et al., 2018; Stilwell, Bahle, & Vecera, 2019; Wang & Theeuwes, 2018a, 2018c; but see Experiment 1 here and Wang & Theeuwes, 2018b). Another explanation for the absence of extra suppression of the low-salience distractor at the high-probability location of the high-salience distractor might be that because a low-salience distractor is generally less distracting, it simply needs less suppression in order to be sufficiently suppressed. In other words, the strength of suppression is not at ceiling but applied suppression is strong enough to effectively suppress a low-salience distractor.

In short, until recently it was unclear whether distractor suppression is an all-or-none phenomenon or adaptive to the strength of suppression needed. Here, we provide strong evidence that the more salient a distractor is, the more suppression is applied.

### Open practices statement

Data and analysis materials for all experiments are available at https://osf.io/wnfbj/ or directly at https://github.com/MichlF/project-MoreCaptureMoreSuppression
